# Does a walking intervention work equally in low and high walkable areas? A large-scale incentive program in Japan

**DOI:** 10.1186/s12966-026-01946-5

**Published:** 2026-07-08

**Authors:** Takumi Abe, Takemi Sugiyama, Neville Owen, Kimihiro Hino

**Affiliations:** 1 School of Commerce, Meiji University, Eifuku 1-9-1, Suginami, Tokyo, 168-8555 Japan; 2 Research Team for Social Participation and Healthy Aging, Tokyo Metropolitan Institute for Geriatrics and Gerontology, Tokyo, Japan; 3 School of Health Sciences, Swinburne University of Technology, Melbourne, VIC Australia; 4 Department of Preventive Medicine and Public Health, Tokyo Medical University, Tokyo, Japan; 5 Baker Heart & Diabetes Institute, Melbourne, VIC Australia; 6 Department of Urban Engineering, Graduate School of Engineering, The University of Tokyo, Tokyo, Japan

**Keywords:** Adherence, Built environment, Physical activity, Step counts, Walkability, Withdrawal

## Abstract

**Background:**

Research investigating whether walking promotion programs work equally at different levels of walkability needs to be scaled up. This study examined the associations of neighbourhood walkability attributes with adult participants’ withdrawal from an incentive-based walking promotion program, and changes in step counts among non-withdrawing participants.

**Methods:**

We used data from 30,530 participants aged 40–74 years enrolled in an incentive walking program in Yokohama City, Japan, in which uploading step-count data was required to enter a raffle for rewards. The follow-up period was up to 48 months. Withdrawal was defined as not uploading step counts during the last six months of the follow-up. Neighbourhood walkability attributes included population density, intersection density, destination diversity, and their composite measure (the Japan Postcode-level Walkability Index). We fitted multilevel Cox proportional hazards models to examine the associations between walkability attributes and program withdrawal, and mixed-effects models to examine the interaction between walkability attributes and time on step counts.

**Results:**

During the follow-up period (median: 28 months), 62% of participants withdrew from the program. Compared to those living in the lowest intersection density areas, those in areas with the highest, the second-highest, and the third-highest intersection density had 7% (95% CI: 0.88, 0.98), 6% (0.89, 0.99), and 7% (0.89, 0.98) lower hazard ratios of withdrawal from the program, respectively. The third-highest population density areas showed a 5% (1.00, 1.10) higher hazard ratio of withdrawal, relative to the lowest. Step counts, which exceeded 7,000 steps/day during the study period, declined between the first and second years but slightly increased from the second to third year. There were no statistically significant interactions between the neighbourhood walkability attributes and time in relation to changes in step counts.

**Conclusion:**

Living in areas with higher street connectivity was associated with a higher retention in the program, whereas the program showed almost comparable effectiveness regarding changes in step counts for continuing participants living in areas with different levels of walkability. These findings suggest the need for initiatives to support adherence to walking-incentive programs, particularly among those who live in areas with less connected streets.

**Supplementary Information:**

The online version contains supplementary material available at https://doi.org/10.1186/s12966-026-01946-5.

## Background

Physical activity confers multiple health benefits, such as reducing the risk of cardiovascular diseases and mortality [1, 2]. Despite the accumulation of such evidence, insufficient physical activity is common, and increasing the level of physical activity is a priority for population health [3]. When considering strategies to promote physical activity, focusing on walking has some advantages: it requires no specific equipment, is regularly performed for leisure and transportation, and is the most prevalent form among various physical activity types (e.g., cycling and sports) [4, 5]. Thus, encouraging people to walk can be a potential action to increase physical activity.

An effective strategy to promote walking is to provide financial incentives [6, 7]. A meta-analysis of 12 randomised controlled trials (RCTs) found that financial incentives had positive effects on daily step counts: they increased by 607 and 514 steps/day during the intervention and post-intervention periods, respectively [8]. Similarly, the effectiveness of financial incentives has been found in observational studies where participants are typically monitored for a longer duration than RCTs. For instance, a 12-month quasi-experimental study using a mobile phone app, in which participants received digital incentives by engaging in healthy behaviours, found an increase in step counts among those who frequently used the app [9]. A three-year observational study in Japan also found that a walking program with incentives, including a chance to receive gift certificates, helped participating older adults to keep the recommended step counts in Japan [10]. However, a considerable proportion of participants dropped from these programs. In the mobile phone study, 40% of the participants for the analysis stop using the app regularly (i.e., using of the app less than 24 weeks out of 52 weeks) [9]. The Japanese study also reported that approximately three-fourths of the sample did not have eligible step counts data (i.e., ≥ 16 days/month of data collection) at the follow-up [10]. Such a large attrition rate is a major threat to the efficacy of the program. In addition, if the rate of attrition differs between areas, the effect of interventions with financial incentives can be uneven, which could unintentionally widen the disparity in physical activity between them.

Research has shown consistently that built environment attributes are associated with physical activity [11, 12]. The built environment is relevant not only to physical activity participation but also to the way physical activity interventions work [13]. A few studies have examined the relationship between built environmental attributes and the adherence of participation in a program. For instance, a US study of a 12-month walking intervention including 252 participants found that the availability of indoor walking facilities (public recreation centres with a treadmill or indoor track in the neighbourhood and shopping malls within 5 miles of home) was associated with a higher level of adherence during the first six months of the walking program [14]. On the other hand, a Canadian study using an internet-delivered 12-week walking intervention with 466 participants found that adherence to the intervention was unrelated to neighbourhood walkability (Walk Score) [15]. Accumulating from diverse settings is needed to advance understanding about whether walking interventions work equally in different contexts. In particular, findings from non-North American research with a larger sample size and longer follow-up period (> 1 year) can further strengthen the evidence base on this topic.

If an intervention works better (i.e., more residents keep participating in the program) in walkable areas where residents are likely to be already active, the intervention may end up widening the gap in physical activity levels between walkable and non-walkable areas. To prevent walking intervention programs from unintentionally expanding disparities in walking, it is important to make sure that such interventions work equally both in low and high walkable areas. Using longitudinal data collected from a large walking incentive program in Japan, we examined the relationship between neighbourhood walkability and its components and withdrawal from the program among adult participants. Additionally, we examined changes in step counts among non-withdrawing participants living in areas with different walkability levels to identify whether neighbourhood walkability influences the long-term effect of the program.

## Methods

### Data source and participants

We used data from the Yokohama Walking Point Program (YWPP). The details of the YWPP have been described previously [10]. Briefly, the YWPP was implemented by the City of Yokohama in November 2014 in Yokohama City, Kanagawa Prefecture, Japan, with the aim of health promotion through daily walking. The YWPP initially targeted adults aged ≥ 40 years living in and commuting to Yokohama. Participants were asked to provide their postal code, date of birth, and gender upon registration for the YWPP. A pedometer was provided to each participant of the YWPP. They were asked to upload their step count data by holding their pedometers over data readers placed in commercial centres, municipal facilities, and other publicly accessible locations. Participants were given incentive points (1 point was awarded for every 1,000 steps up to 10 points per day), which allowed them to enter a raffle for rewards (e.g., gift cards and health equipment). Winners were announced every three months, with higher points leading to greater chances to receive rewards.

The baseline for this study was April 2016, after when the YWPP expanded its effort to recruit participants through involving workplaces as well as increasing the number of data readers. The number of locations equipped with data readers increased to over 1,000 before April 2016, covering approximately 90% of post codes. The number of adults (aged 40–74 years) who registered for the YWPP between April 2016 and March 2017 was 46,327. The study protocol was approved by the Ethical Committee of School of Engineering, The University of Tokyo (approval number KE20-56).

### Outcomes

The primary outcome was withdrawal from the YWPP. The follow-up period of this study was until March 2020, as the state of emergency for COVID-19 was declared in April 2020 in Yokohama City. Withdrawal was defined as not uploading their step count between October 2019 and March 2020 (i.e., the last six months of the follow-up period). When participants met the event definition, the time-to-event was defined as the interval from baseline to the month in which the last data were uploaded.

The secondary outcome was the change in step counts of those who did not withdraw from the YWPP during the study period. We collected monthly pedometer-based step count data, along with the number of days participants wore their pedometers each month. Valid pedometer data (≥ 16 days/month) [10] were used to calculate daily steps for each month and were then transformed into annual average number of steps (steps/day). In this estimation, the participants were eligible if they had valid data at least once for one month in the first year. The annual average number of daily steps was capped at 30,000 steps per day for values ≥ 30,000, while daily step counts below 1,000 were treated as invalid data due to insufficient pedometer wear time [16, 17].

### Exposures

Neighbourhood walkability attributes were assessed at the postal code level. There are 760 post codes in the study area, with the median population of 3,070. We used the Japan Postcode-level Walkability Index (JPWI), which was developed to enable assessment of walkability across Japan, using data from 113,156 postcodes nationwide [18, 19]. It is composed of population density, intersection density, and destination diversity, which were assessed within a 1-km network buffer drawn from the centroid of the 250-m mesh block with the highest population within each postal code area. For each postcode, population density was calculated by dividing the population count within the buffer based on the 2020 Japanese census by the buffer area. Intersection density was estimated by dividing the number of intersections within the buffer by the area of the buffer. Destination diversity was measured by counting the presence of 18 types of daily-life facilities (e.g., supermarkets, pharmacies, banks) within the buffer, resulting in a score ranging from 0 to 18. ArcGIS Geo Suite 2021 was used for the calculation of intersection density, while the National Land Numerical Information provided by the Ministry of Land, Infrastructure, Transport and Tourism of Japan and the 2021 Nippon Telegraph and Telephone Corporation telephone directory were used to identify those facilities. The three components were first square-root transformed to reduce skewness and then standardised across all the post codes in Japan (converted to z-scores). Finally, the sum of the three z-scores was divided by three to calculate the JPWI. The median (interquartile range) of the JPWI across Japan was 0.0 (-0.8, 0.8). It has been confirmed that higher JPWI is associated with longer walking time [18].

### Statistical analysis

A retention curve was depicted to show the proportion of participants who continued to participate in the YWPP during the follow-up period. We fitted multilevel Cox hazard population models to examine the relationship of withdrawal from the YWPP with the JPWI and its components. Postal code level clustering was taken into account by including the random intercept for postal codes in the models. JPWI and each built environment attribute was independently divided into quartiles (Q1: least walkable; Q4: most walkable) for these analyses to facilitate interpretation and ensure a sufficient sample size in each category. Each environmental attribute was separately examined in the models. There were two regression models. Model 1 adjusted for gender, age, and registration month to control for seasonal effects, while Model 2 further adjusted for area-level socioeconomic status at municipality level. For area-level socioeconomic status, we employed the area deprivation index (ADI) in Japan, which consisted of household composition, housing, and labour and occupational status derived from the 2015 census [20]. The 2015 ADI data were allocated to 1,886 municipalities and administrative ward across Japan (median [interquartile range]: 6.0 [5.6, 6.5]), with higher scores indicating greater deprivation. In Yokohama City, the ADI data were assigned to 18 administrative wards (median: 5.2 [4.9, 5.4]).

We conducted a sensitivity analysis restricting the follow-up period to 1 year, in which withdrawal was defined as not uploading step count data at 12 months after baseline (i.e., initial registration for the YWPP). This analysis was performed to examine whether the association was observed during the early stage of participation in the program.

A mixed-effects model was used to examine changes in step counts among non-withdrawing participants during the three years. The model included each neighbourhood walkability attribute, a squared time variable (which provided a better fit than a non-squared version), their interaction, and the same covariates for in Model 2 as fixed effects. Individuals were treated as a random effect. All analyses were performed by Stata version 17 (Stata Corp., College Station, TX, USA) with a significant level of 0.05.

## Results

### Characteristics of the study participants and the rate of withdrawal

**Fig. 1:**
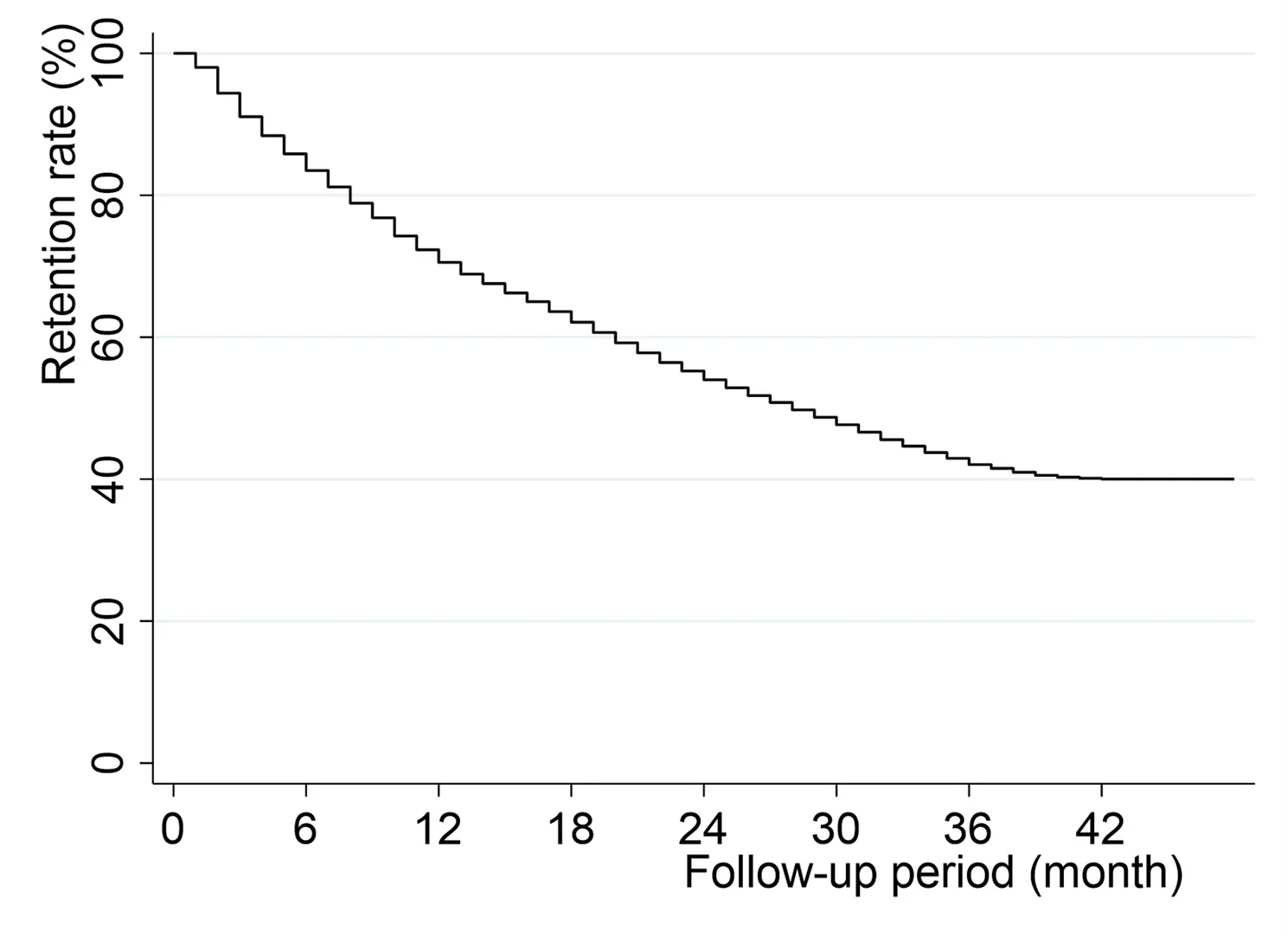
Retention curve

The number of eligible participants was 30,530, after excluding those who resided outside Yokohama City (*n* = 3,562) and those who had never uploaded their step counts (*n* = 12,235). Table 1 shows the characteristics of the participants who remained in the program and those who withdrew from the program. Women (58%) and middle-aged adults between 40 and 64 years (70%) were the majority of the sample. During the follow-up period (median: 28 months), 62% of them withdrew from the YWPP. Figure 1 shows the retention curve. The number of withdrawals was larger initially but decreased gradually over time. Approximately 17% of participants withdrew from the YWPP within the first 6 months, and additional 12% in the next 6 months.

**Table 1:** Characteristics of participants % or median (1Q, 3Q)

	Non-withdrawals (*n* = 12,299)	Withdrawals (*n* = 18,231)	All (*n* = 30,530)
Women	53.0	60.6	57.6
Older adults ≥ 65 years	37.3	25.5	30.2
ADI	5.2 (4.9, 5.4)	5.2 (4.9, 5.4)	5.2 (4.9, 5.4)
JPWI	1.3 (1.1, 1.5)	1.3 (1.1, 1.5)	1.3 (1.1, 1.5)
Population density, n/ha	115.8 (93.4, 143.7)	116.9 (95.5, 145.8)	116.4 (95.0, 145.0)
Intersection density, n/ha	2.1 (1.8, 2.5)	2.1 (1.7, 2.5)	2.1 (1.7, 2.5)
Destination diversity^a^, n	15 (13, 16)	15 (13, 16)	15 (13, 16)

^a^ The number of destination types (range: 0–18) within the 1,000 m network distance buffer drawn from the centroid of the 250-m mesh block with the highest population within each postal code area

*ADI* Area Deprivation Index, *JPWI* Japan Postcode-level Walkability Index, *1Q* First quartile, *3Q* Third quartile

There were no substantial differences in walkability or its components between participants who withdrew from the program and those who did not. Supplementary Table 1 shows the characteristics of the study areas and their neighbourhood walkability. Among 760 postal codes in Yokohama, 45 postal codes had no residents, and 16 had no study participants, resulting in 699 postcode areas. The median number of participants per postal code was 26 at baseline (interquartile range: 12, 61). Supplementary Table 2 shows correlations among built environment attributes. JPWI components showed moderate intercorrelations.

### Association of withdrawal from the program with walkability and its components

Table 2 shows the association of withdrawal from the YWPP with neighbourhood walkability and its components. In Model 1, the second-highest population density area (Q3) was associated with a higher hazard ratio for YWPP withdrawal relative to the lowest density area (Q1). Compared with the lowest intersection density area, areas with higher intersection density were associated with lower hazard ratios for withdrawal from the YWPP. These associations remained in Model 2: those in the second-highest population density area had a 5% (95% CI: 1.00, 1.11) higher hazard ratio for YWPP withdrawal, whereas those living in the highest, the second-highest, and the third-highest intersection density areas showed 7% (95% CI: 0.88, 0.98), 6% (0.89, 0.99), and 7% (0.89, 0.98) lower hazard ratios for withdrawal from the YWPP. There were no significant associations of withdrawal from the YWPP with the JPWI and destination diversity.

In the sensitivity analysis using the shorter follow-up period, the highest and the third-highest intersection density areas showed 12% (0.82, 0.95) and 8% (0.86, 0.98) lower hazard ratios for withdrawal from the YWPP, respectively (Supplementary Table 3). No other walkable attributes were significantly associated with withdrawal from the YWPP in the sensitivity analysis.

**Table 2:** Hazard ratios (95% CI) of withdrawal from the YWPP according to walkability and its components

	Q1	Q2	Q3	Q4
Model 1				
JPWI	Ref.	0.98 (0.94, 1.03)	0.96 (0.92, 1.01)	0.97 (0.92, 1.03)
Population density	Ref.	1.02 (0.98, 1.07)	1.05 (1.00, 1.11)*	1.05 (0.99, 1.11)
Intersection density	Ref.	0.93 (0.89, 0.97)**	0.92 (0.88, 0.97)**	0.91 (0.86, 0.96)***
Destination diversity	Ref.	1.02 (0.97, 1.07)	0.99 (0.95, 1.04)	0.99 (0.94, 1.04)
*Model 2*				
JPWI	Ref.	0.98 (0.94, 1.03)	0.97 (0.93, 1.02)	0.98 (0.93, 1.04)
Population density	Ref.	1.02 (0.98, 1.07)	1.05 (1.00, 1.10)*	1.04 (0.99, 1.10)
Intersection density	Ref.	0.93 (0.89, 0.98)**	0.94 (0.89, 0.99)*	0.93 (0.88, 0.98)**
Destination diversity	Ref.	1.02 (0.97, 1.06)	0.99 (0.95, 1.04)	0.99 (0.94, 1.04)

Model 1 adjusted for age category, gender, and registration month. Model 2 further adjusted for area-level socioeconomic status

*JPWI* Japan Postcode-level Walkability Index

Q1: Least walkable; Q4: Most walkable

* p < .05, ** p < .01, *** p < .001

### Association of step count change with walkability and its components

**Fig. 2:**
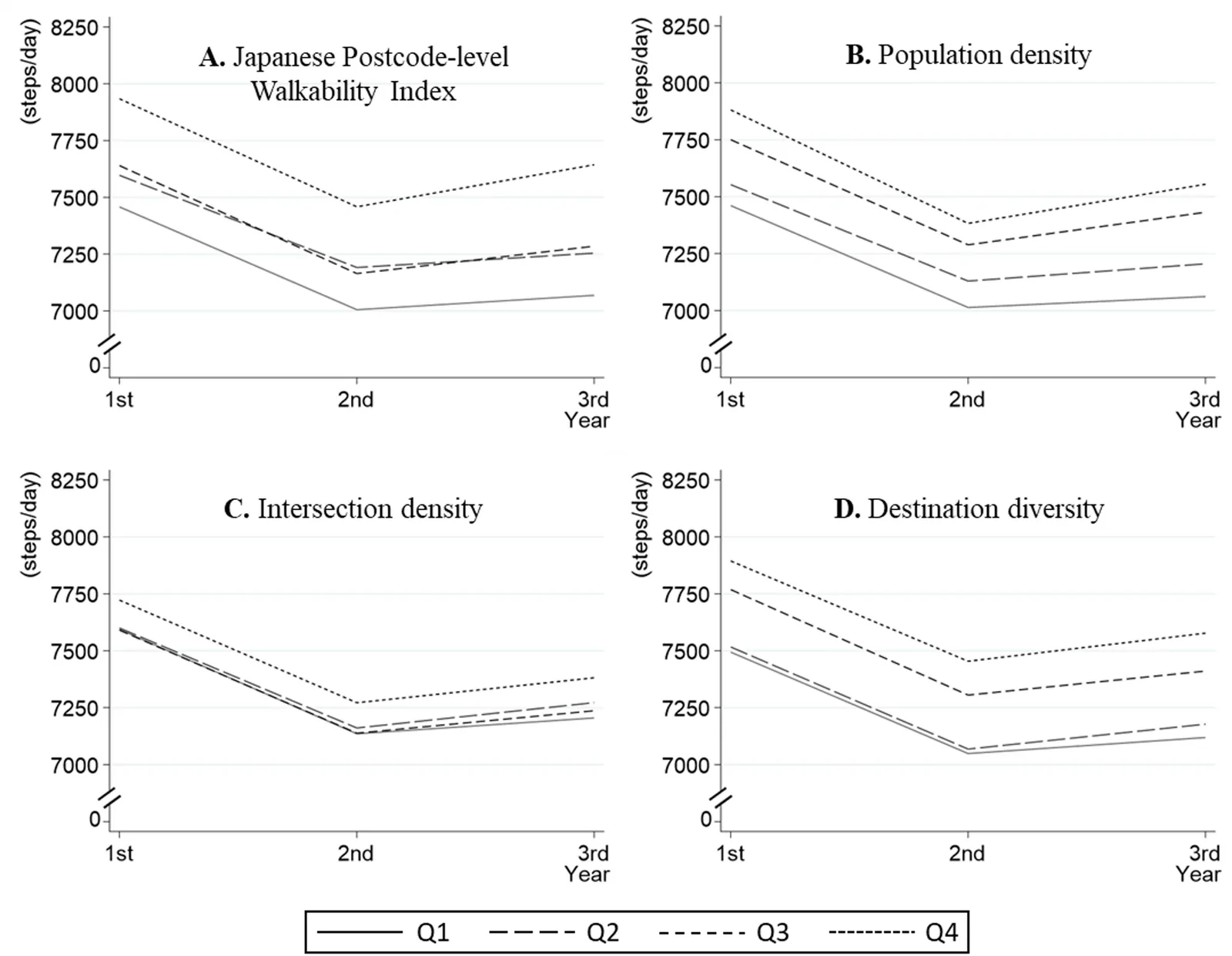
Three-year trends inestimated marginal means of step counts according to (**A**) walkability index, **B** population density, **C** intersection density, and **D** destination diversity. The first quartile (lowest in walkability) is shown as a solid line, the second as a long-dashed line, the third as a dashed line, and the fourth as a short-dashed line

Among participants who remained in the program, 114 did not upload their step count data in the first year, and five had daily step counts below 1,000. Consequently, step counts for three years were estimated using 35,569 yearly records from 12,180 participants. Figure 2 shows the trend in step counts for three years, estimated using the mixed-effects model. Step counts declined between the first and second years, but slightly increased from the second to third year (Supplementary Table 4). This trend was consistent across quartiles of each built environment attribute. No significant interaction was found between the neighbourhood walkability attributes and the squared time variable in any of the models.

## Discussion

There was limited research on how walking programs with incentives work in areas that differ in walkability. Using 4-year follow-up data obtained from 30,530 participants in the city-wide walking program with incentives, we found that living in areas with a higher walkability attribute (intersection density) was associated with less withdrawal from the program, while higher population density may be associated with higher withdrawal. It was also found that the long-term effect of the program among those who remained in the program did not seem to vary by walkability (or its components). These findings suggest that the walking program may have been easier to adhere for areas with better street connectivity.

We found intersection density in the neighbourhood to be associated with withdrawal from the program. This association remained after adjusting for area-level socioeconomic status. Such adjustment could potentially alter the association between walkability attributes and withdrawal from the program because area-level socioeconomic status can be associated with neighbourhood walkability [21, 22]. The 75th percentile of the ADI in our target areas fell below the 25th percentile of the nationwide distribution, indicating that the target areas were relatively affluent. Therefore, area-level socioeconomic status may have had only a small effect on the association, resulting in comparable findings between Models 1 and 2. Areas with more connected streets have some advantages, such as making it easier to walk and reach destinations. Additionally, such areas are likely to have larger footpath areas [23], which contribute to comfortable walking. These combined effects may support long-term engagement with the program. Since these interpretations are speculative, further research is needed for deeper understanding of the role of neighbourhood walkability attributes in retaining participants in programs aiming to increase physical activity.

We found that higher population density was marginally associated with more withdrawal from the program. Although the limited amount of data (e.g., no data regarding individual-level socioeconomic status) in this study makes it difficult to determine the underlying reasons, several possible explanations can be considered. For instance, financial incentives may be less attractive and less effective among those with higher economic status than those with lower economic status [24]. Another possible explanation is that high-density areas may have downsides, such as being more crowded and less pleasant to walk (due to pollution and noise), which could discourage walking. Nonetheless, future studies are needed to clarify the role of population density in this association, accounting for potential confounders.

Step counts decreased over time, especially from the first and second years, among those who did not withdraw from the walking program. This finding suggests that there may have been an initial excitement, but it is challenging to maintain higher step counts over time. In this regard, we observed no significant interaction between walkability attributes and time on step counts, with the similar step count trends across their quartiles. The findings seem to suggest that the walking program worked equally among continuing participants living in areas that differed in walkability, although the initial step count was different between them as shown in Fig. 2. It should be noted that step counts data were retrieved only from those who remained in the program, which may be subject to attrition bias. Additionally, the lack of significant interactive effects between walkability and time on step counts is consistent with previous literature. A 12-week pedometer-facilitated intervention reported no group-by-time interaction (walkable vs. car-dependent areas) on walking time, although those in walkable areas showed longer walking time than those in car dependent area during the intervention period [25]. Taken together with our results, step counts may follow a trend that carries over the initial baseline differences attributable to walkability throughout the intervention period.

In this study, the estimated marginal means of step counts exceeded 7,000 steps/day, which is a clinically meaningful threshold [26], during the follow-up period even among those living in less walkable neighbourhoods in the study areas (Fig. 2, Supplementary Table 4). These step counts are higher than the national average for Japanese adults aged 20–64 years (6,957 steps/day), as reported in a Japanese survey [27]. The findings on step counts suggest that the YWPP was successful for participants to maintain higher levels of walking. However, it should be also highlighted that 60% of the initial participants dropped from the program. Further efforts, ideally targeting areas/groups that tend to have a higher level of attrition such as those living in areas with less connected streets, are needed to increase the effect of the program. Health behaviours are influenced by various internal and external factors that interact in complex ways across multiple levels [28, 29]. Therefore, the negative effects of unfavourable built environment attributes on people’s behaviours may be mitigated by other supportive factors, potentially through strategies such as social campaigns designed to maintain momentum and enhance individuals’ motivation to actively participate in the program.

Results from the sensitivity analysis supported the direction of the association between neighbourhood walkability attributes and withdrawal from the program shown in the primary analysis. Hazard ratios changed slightly in the sensitivity analysis, and no significant association between population density and withdrawal from the program was observed (Supplementary Table 3). These results suggest that the primary findings, especially for intersection density, are confirmed even when focusing on the early period of the follow-up.

This study found that approximately 60% of participants withdrew from the walking program within 4 years. Our finding about the proportion of withdrawal may not be unique. One-year follow-up studies reported adherence rates to physical activity programs being 43% [30] and 60% [9]. This cannot be directly compared with our results due to differences in follow-up methods and outcome definitions (adherence and withdrawal). However, this study is distinct from the previous studies that focused on adherence throughout the period of intervention [14, 30], since we monitored when participants withdrew from the program. We found that the rate of withdrawal from the YWPP gradually decreased over the 4-year follow-up period. There would be larger dropouts at the initial stage of a program, as some program participants who find its requirements too hard to follow (e.g., conflicting with their life pattern) are likely to give up quickly.

This study has several strengths, such as large sample size, long follow-up period and the use of objective data (uploading pedometer data, measured step counts) for the outcomes. This study also has several limitations. This study also has several limitations. The JPWI was a composite of environmental attributes measured in and after 2020, which is the end of the follow-up period. Although environmental attributes tend to be stable over several years, some post code areas may have experienced considerable environmental changes (e.g., redevelopment) during the follow-up period, which may have affected our results. We did not examine some potentially relevant environmental attributes, such as accessibility to public transportation, traffic conditions and green space availability. This limitation arose from the use of the JWPI and its components, which enabled us to employ standardised walkability measures developed for Japan but which does not include these other attributes. In addition, we did not capture changes in neighbourhood walkability attributes. These other attributes should be considered in future studies. Although our primary outcome was withdrawal from the program, we also examined step count change. Since there were no step count data before the program, it was not possible for us to compare step counts before and after the intervention. Some participants were unable to maintain their participation due to relocation from Yokohama city. This may have inflated the percentage of withdrawal, although the rate of migration out of Yokohama city is low (around 3–4% annually). The use of data obtained from a single city makes it difficult to generalise the findings to other localities. Although the city of Yokohama is large and included a wide range of areas from urban to suburban, most of them were walkable (relatively high in the densities of population and intersections as shown in Supplementary Table 1). Consequently, our results may not be applicable to areas with lower walkability. Nevertheless, it should be noted that we did find variations in the rate of withdrawal between areas of lower and higher intersection density, suggesting the relevance of the variability of these attributes.

## Conclusions

This longitudinal study highlights the potential of neighbourhood walkability attributes in affecting adherence to walking programs with incentives. Our findings suggest that the program may not be easy to follow for residents of areas with higher population density and lower intersection density. However, among those who kept uploading the pedometer data, the program appeared to have similar effects across areas with different levels of walkability attributes, enabling them to maintain on average 7,000 steps/day over the 3-year period. These findings imply that further support for residents living in such areas may be needed to reduce program withdrawal and thereby enhance program effectiveness. Future research is warranted to understand why these areas were higher in withdrawal and identify ways to improve program adherence in such areas.

## Supplementary Information

Supplementary Material 1.

## Data Availability

The data that support the findings of this study are not openly available due to a confidentiality agreement with survey respondents.

## Abbreviations

ADI: Area deprivation index
CI: Confidence interval
GIS: Geographic Information System
JPWI: Japan Postcode-level Walkability Index
RCT: Randomised controlled trial
YWPP: Yokohama Walking Point Program
