# Supplementary material for: Does a walking intervention work equally in low and high walkable areas? A large-scale incentive program in Japan
Source: Int J Behav Nutr Phys Act. 2026 Jul 8;23:88. doi: 10.1186/s12966-026-01946-5 (PMC13632372; doi:10.1186/s12966-026-01946-5)
Supplement: Supplementary file 1 [file 12966_2026_1946_MOESM1_ESM.docx]

Supplementary Table 1. Characteristics of the study area according to the Japanese Postcode-level Walkability Index

|  | Q1 | Q2 | Q3 | Q4 | Total |
| --- | --- | --- | --- | --- | --- |
| Mean % of withdrawal | 62.5 | 61.5 | 61.3 | 61.5 | 61.7 |
| Median JPWI (1Q, 3Q) | 0.8 (0.7, 1.0) | 1.2 (1.1, 1.3) | 1.5 (1.4, 1.5) | 1.8 (1.7, 1.9) | 1.3 (1.1, 1.6) |
| N (post code areas) | 175 | 175 | 175 | 174 | 699 |
| Mean geographic size ± SD, ha | 89.5 ± 82.1 | 67.2 ± 59.0 | 54.4 ± 52.7 | 19.5 ± 25.7 | 57.7 ± 63.6 |
| ADI | 5.2 (4.4, 5.3) | 5.2 (4.9, 5.3) | 5.2 (5.0, 5.4) | 5.1 (5.0, 5.3) | 5.1 (4.9, 5.3) |
| N (participants) | 7,774 | 8,989 | 9,466 | 4,301 | 30,530 |
| Median population density (1Q, 3Q), n/ha | 80.4 (66.7, 95.1) | 107.4 (95.5, 123.8) | 139.5 (120.4, 155.9) | 183.9 (168.2, 203.6) | 124.3 (95.1, 162.7) |
| Median intersection density (1Q, 3Q), n/ha | 1.5 (1.3, 1.8) | 1.9 (1.7, 2.2) | 2.2 (2.1, 2.6) | 2.9 (2.6, 3.0) | 2.1 (1.7, 2.6) |
| Median destination diversity^a^ (1Q, 3Q), n | 11 (9, 13) | 15 (13, 16) | 16 (15, 16) | 17 (16, 17) | 15 (13, 16) |

^a^ The number of destination types (range: 0–18) within the 1,000 m network distance buffer drawn from the centroid of the 250-m mesh block with the highest population within each postal code area.

ADI: Area Deprivation Index; JPWI: Japan Postcode-level Walkability Index; 1Q: First quartile; 3Q: Third quartile

Q1: Least walkable; Q4: Most walkable

Supplementary Table 2. Pearson’s correlation coefficients between built environment attributes (N=699)

|  | JPWI | Population density | Intersection density |
| --- | --- | --- | --- |
| JPWI |  |  |  |
| Population density | 0.90 |  |  |
| Intersection density | 0.82 | 0.58 |  |
| Destination diversity | 0.77 | 0.61 | 0.47 |

p < .001 for all correlations.

JPWI: Japan Postcode-level Walkability Index

Supplementary Table 3. Hazard ratios (95% CI) of withdrawal within 1 year from the YWPP according to built environment attributes

|  | Q1 | Q2 | Q3 | Q4 |
| --- | --- | --- | --- | --- |
| *Model 1* |  |  |  |  |
| JPWI | Ref. | 1.00 (0.94, 1.06) | 0.95 (0.89, 1.01) | 0.97 (0.90, 1.04) |
| Population density | Ref. | 1.00 (0.94, 1.07) | 1.03 (0.97, 1.10) | 1.02 (0.94, 1.10) |
| Intersection density | Ref. | 0.92 (0.86, 0.98)** | 0.94 (0.88, 1.01) | 0.88 (0.82, 0.95)*** |
| Destination diversity | Ref. | 1.01 (0.95, 1.07) | 0.99 (0.93, 1.06) | 0.98 (0.91, 1.05) |
| *Model 2* |  |  |  |  |
| JPWI | Ref. | 1.00 (0.94, 1.06) | 0.96 (0.90, 1.02) | 0.98 (0.90, 1.05) |
| Population density | Ref. | 1.00 (0.94, 1.07) | 1.03 (0.96, 1.10) | 1.01 (0.93, 1.09) |
| Intersection density | Ref. | 0.92 (0.87, 0.98)* | 0.96 (0.90, 1.03) | 0.91 (0.84, 0.97)** |
| Destination diversity | Ref. | 1.00 (0.95, 1.07) | 0.99 (0.93, 1.06) | 0.98 (0.91, 1.05) |

** *p* < .01, *** *p* < .001

Model 1 adjusted for age category, gender, and registration month. Model 2 further adjusted for area-level socioeconomic status.

JPWI: Japan Postcode-level Walkability Index

Q1: Least walkable; Q4: Most walkable

Supplementary Table 4. Estimated marginal means of step counts (95% CI) per day according to quartiles of built environment attribute

|  | 1st year | 2nd year | 3rd year |
| --- | --- | --- | --- |
| JPWI |  |  |  |
| Q1 | 7458.1 (7353.2, 7563.0) | 7005.5 (6900.2, 7110.9) | 7068.7 (6963.3, 7174.1) |
| Q2 | 7597.1 (7499.6, 7694.6) | 7191.3 (7093.4, 7289.1) | 7254.5 (7156.5, 7352.5) |
| Q3 | 7640.0 (7545.2, 7734.7) | 7164.7 (7069.4, 7260.0) | 7285.1 (7189.7, 7380.6) |
| Q4 | 7934.0 (7792.6, 8075.3) | 7458.4 (7316.6, 7600.3) | 7643.6 (7501.5, 7785.7) |
| Population density |  |  |  |
| Q1 | 7461.0 (7357.6, 7564.4) | 7013.6 (6909.8, 7117.5) | 7061.6 (6957.7, 7165.5) |
| Q2 | 7553.1 (7464.1, 7642.1) | 7129.8 (7040.4, 7219.2) | 7205.4 (7115.8, 7294.9) |
| Q3 | 7750.3 (7647.0, 7853.7) | 7288.8 (7184.8, 7392.8) | 7431.9 (7327.8, 7536.0) |
| Q4 | 7881.2 (7732.9, 8029.5) | 7383.1 (7234.2, 7531.9) | 7554.9 (7405.8, 7703.9) |
| Intersection density |  |  |  |
| Q1 | 7593.8 (7484.7, 7702.9) | 7135.5 (7026.0, 7245.1) | 7205.0 (7095.3, 7314.6) |
| Q2 | 7599.9 (7506.1, 7693.7) | 7161.0 (7066.8, 7255.2) | 7272.2 (7177.9, 7366.6) |
| Q3 | 7590.4 (7482.2, 7698.7) | 7138.2 (7029.4, 7246.9) | 7236.7 (7127.9, 7345.5) |
| Q4 | 7722.4 (7607.5, 7837.2) | 7271.8 (7156.4, 7387.1) | 7381.4 (7265.9, 7496.9) |
| Pedestrian street density |  |  |  |
| Q1 | 7494.0 (7401.6, 7586.4) | 7048.9 (6956.0, 7141.7) | 7119.1 (7026.2, 7212.1) |
| Q2 | 7516.7 (7421.6, 7611.8) | 7068.4 (6972.8, 7164.0) | 7177.9 (7082.2, 7273.6) |
| Q3 | 7768.6 (7652.4, 7884.7) | 7305.4 (7188.7, 7422.0) | 7410.9 (7294.0, 7527.8) |
| Q4 | 7894.4 (7763.0, 8025.7) | 7454.1 (7322.1, 7586.1) | 7577.2 (7445.1, 7709.3) |

Models adjusted for age category, gender, registration month, and area-level socioeconomic status.

Q1: Least walkable; Q4: Most walkable
